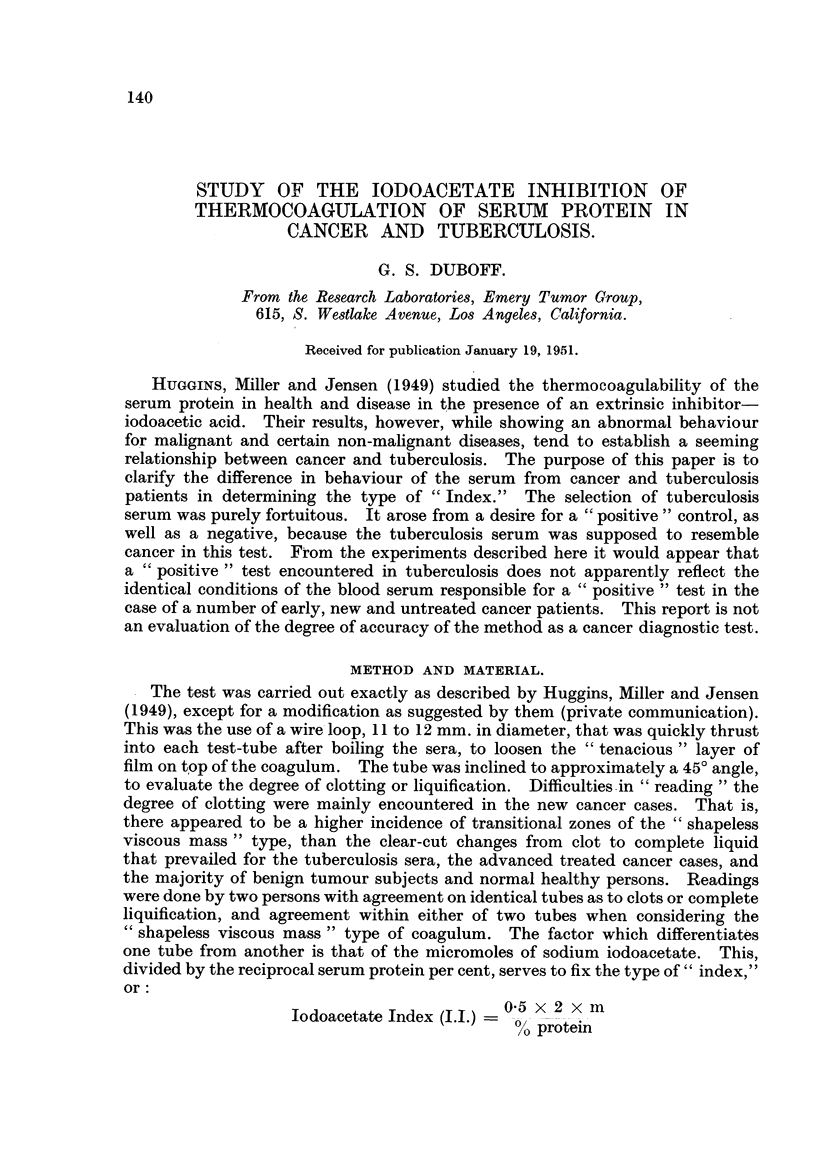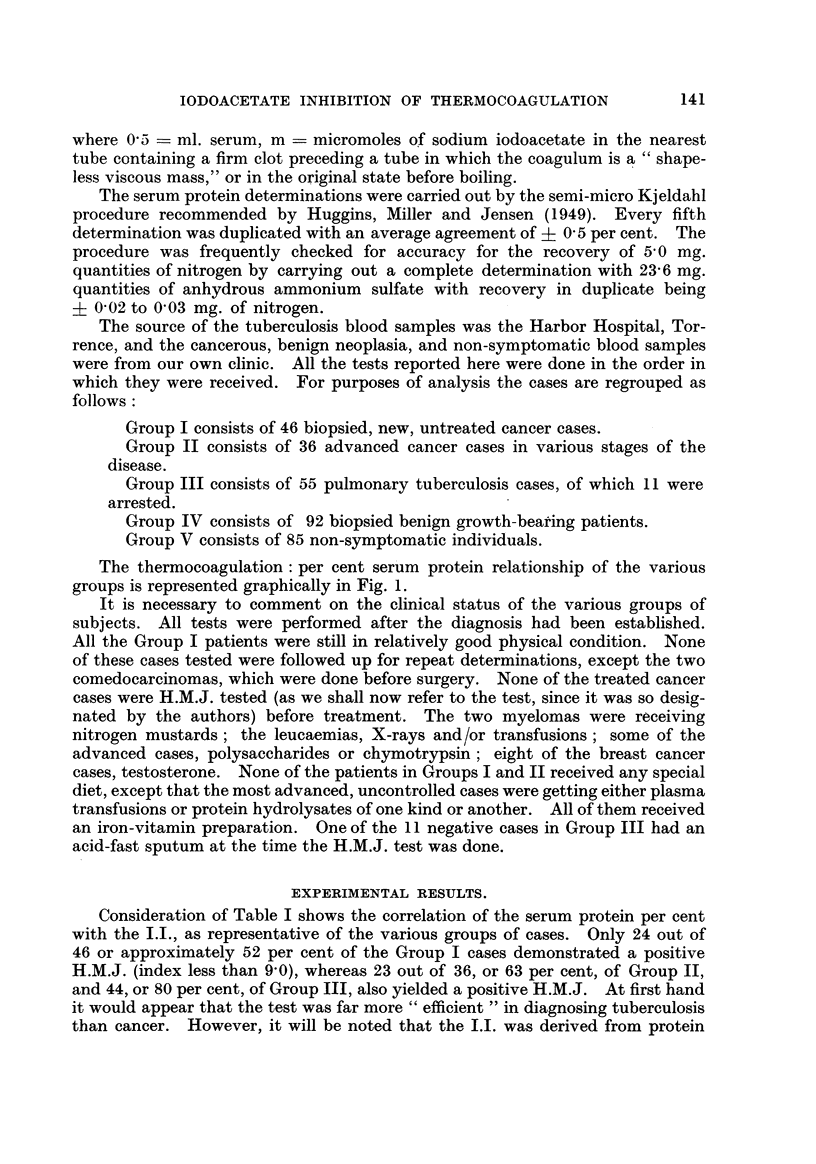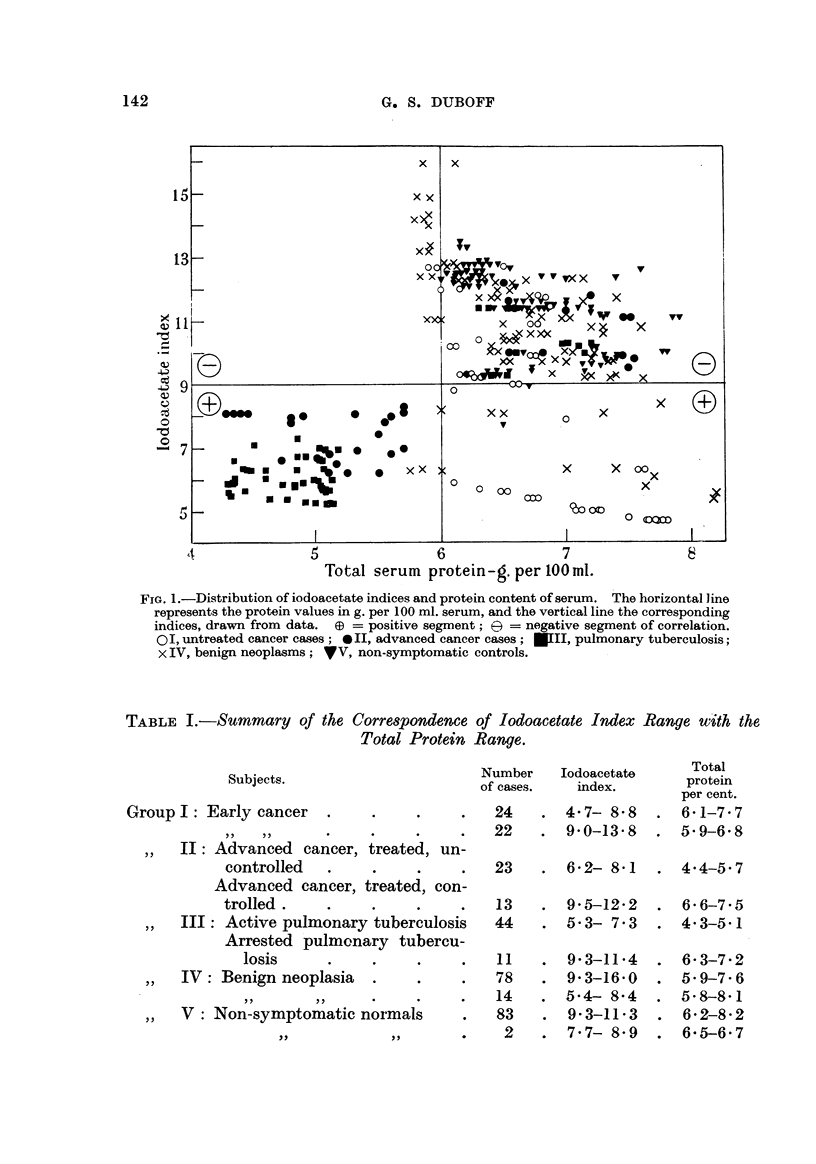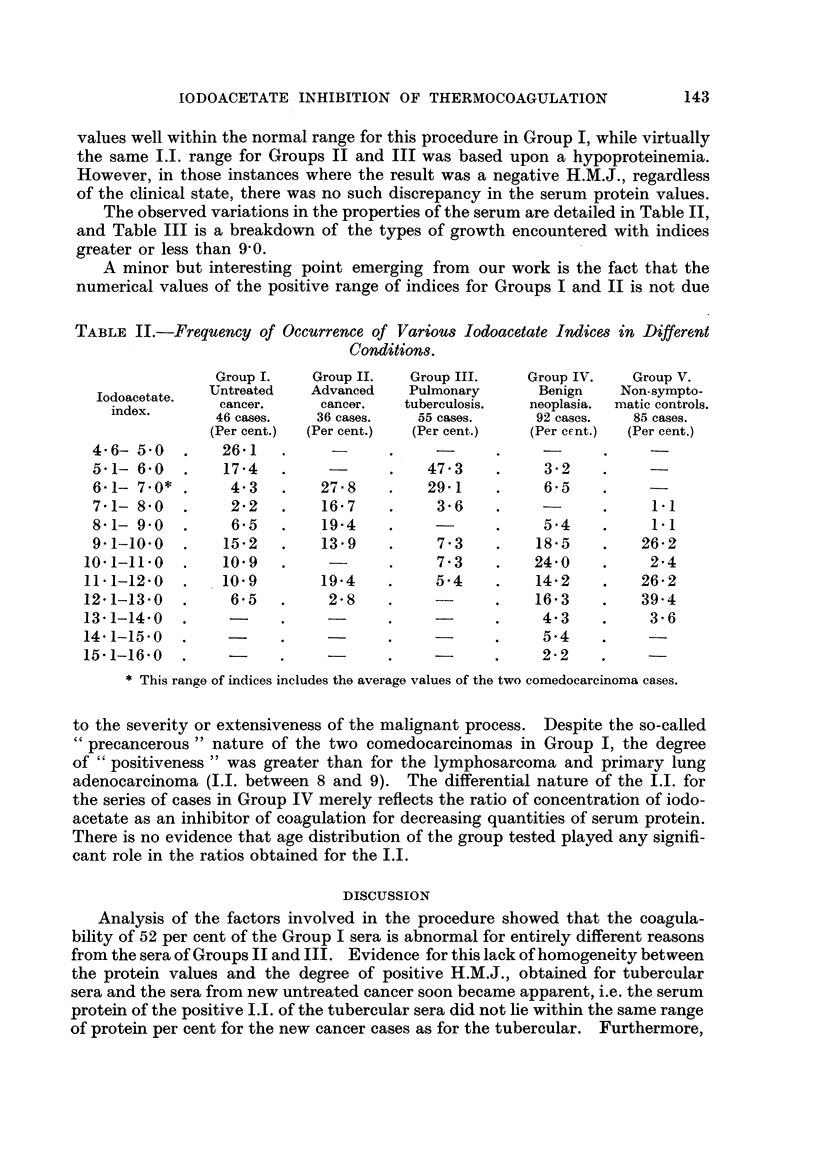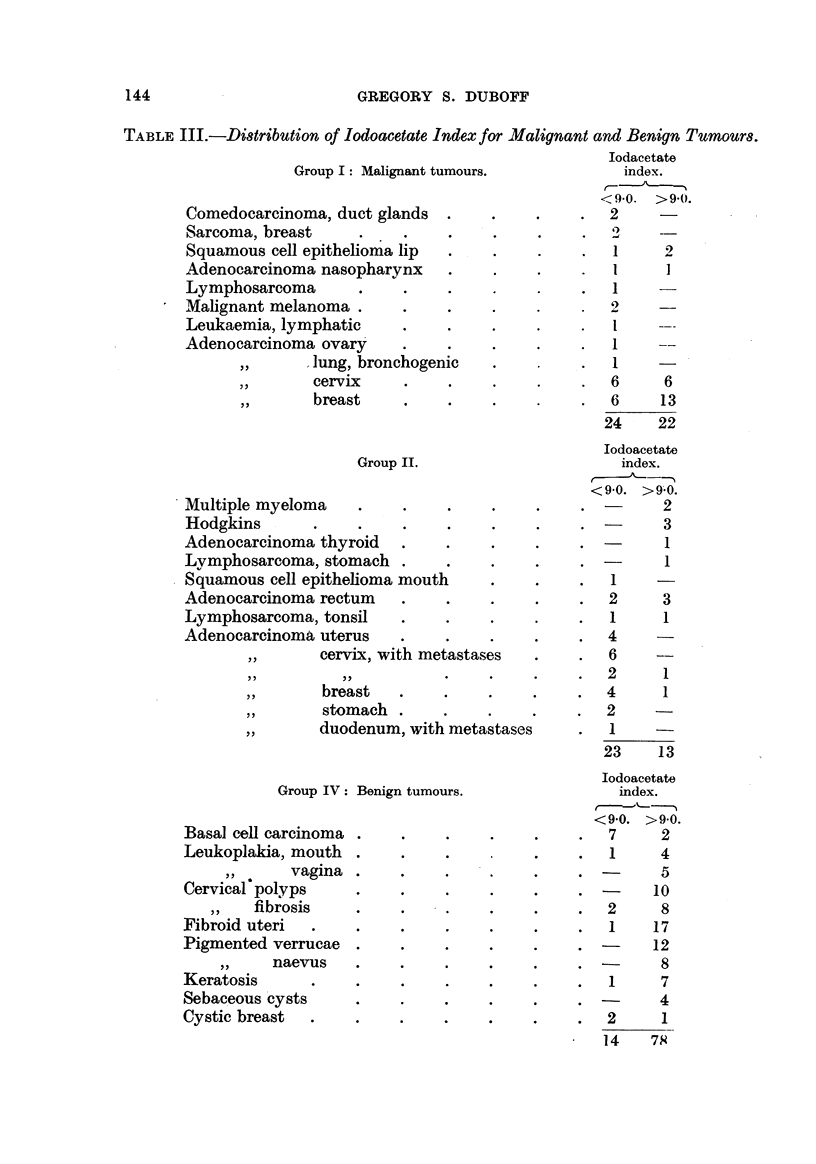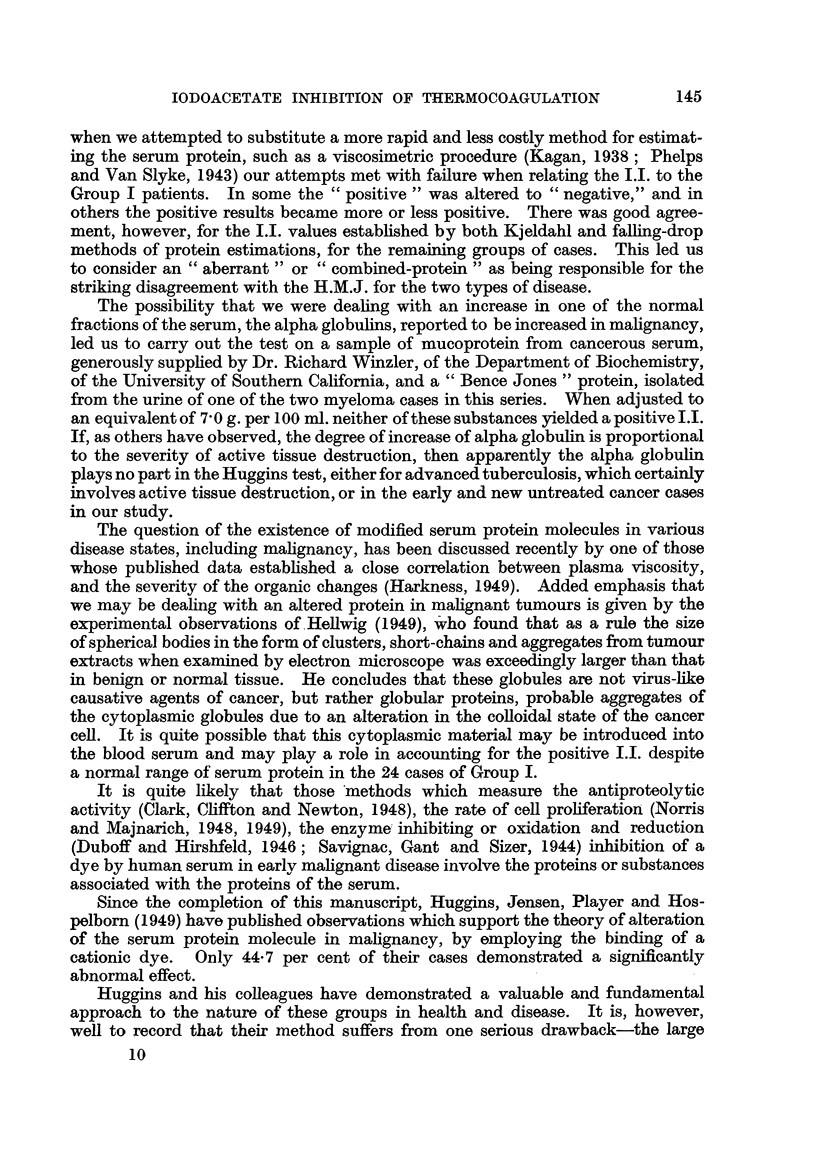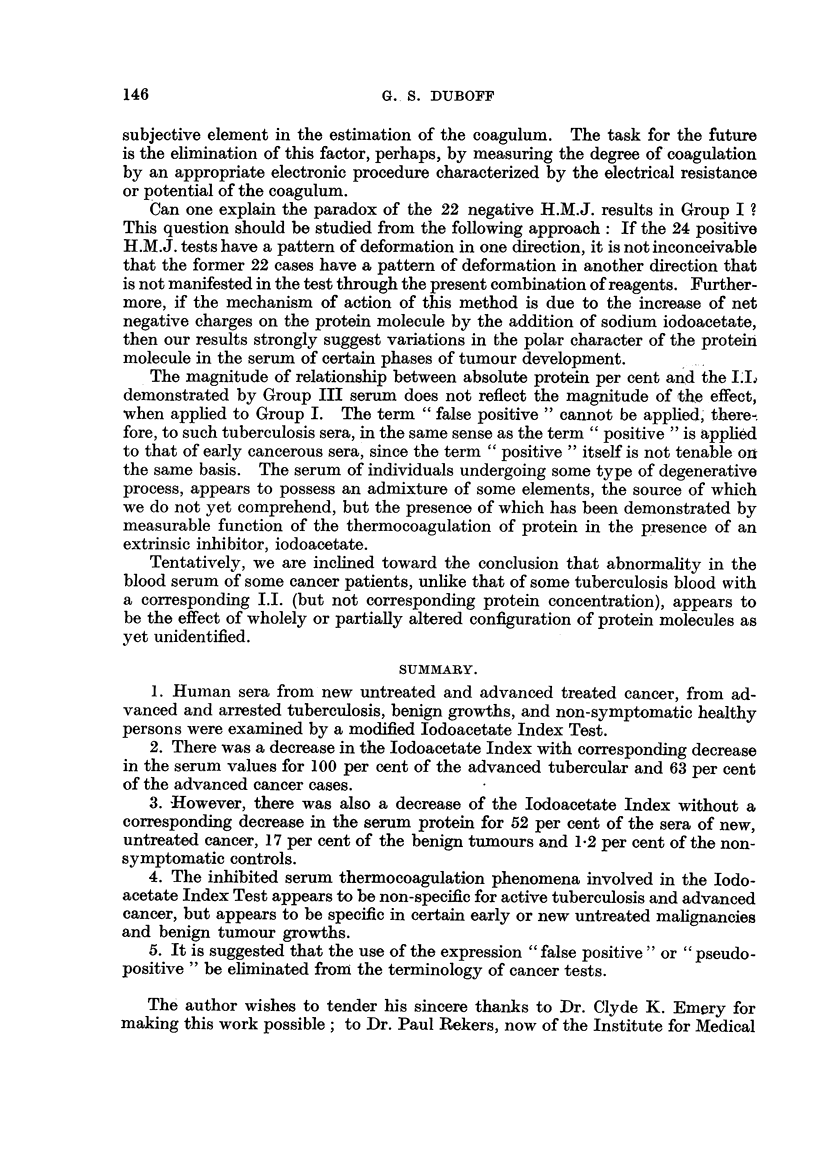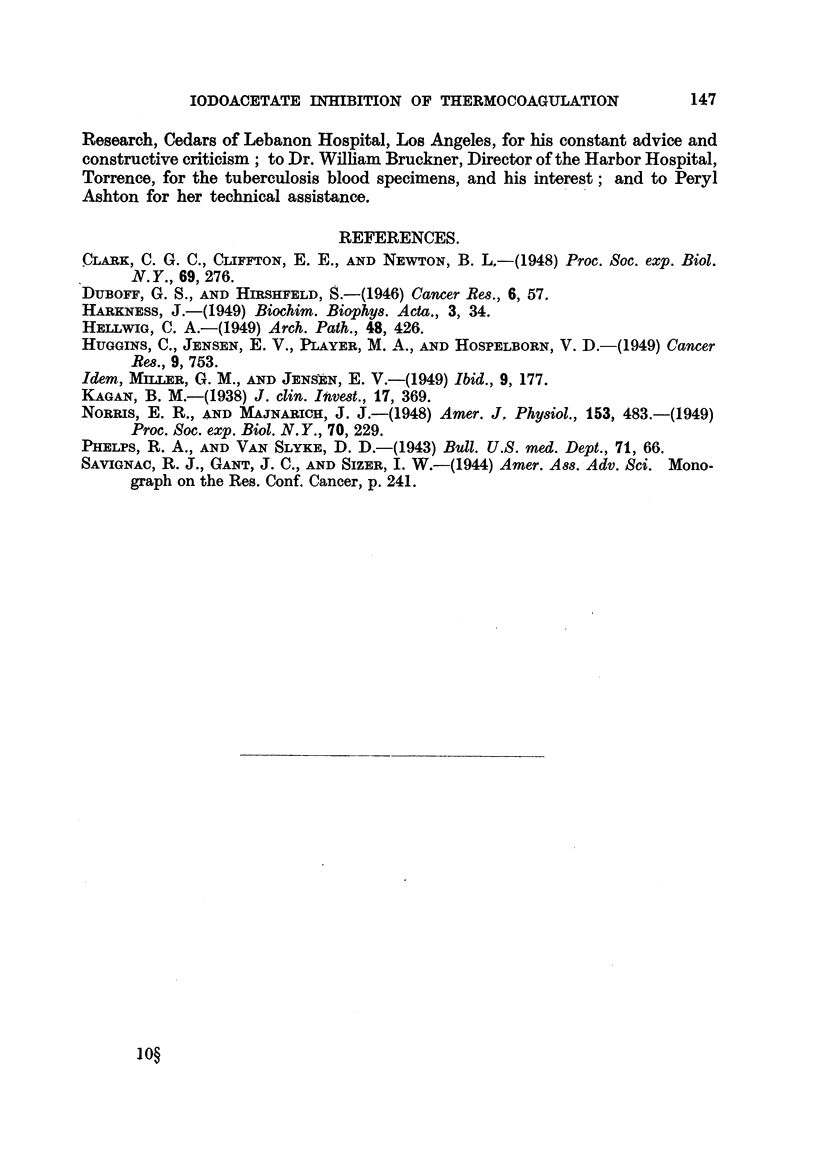# Study of the Iodoacetate Inhibition of Thermocoagulation of Serum Protein in Cancer and Tuberculosis

**DOI:** 10.1038/bjc.1951.14

**Published:** 1951-03

**Authors:** G. S. Duboff


### STUDY OF THE IODOACETATE INHIBITION OF THERMOCOAGULATION OF SERUM PROTEIN IN CANCER AND TUBERCULOSIS.

G. S. DUBOFF.

*From the Research Laboratories, Emery Tumor Group, 615, S. Westlake Avenue, Los Angeles, California.*

Received for publication January 19, 1951.

HUGGINS, Miller and Jensen (1949) studied the thermocoagulability of the serum protein in health and disease in the presence of an extrinsic inhibitor—iodoacetic acid. Their results, however, while showing an abnormal behaviour for malignant and certain non-malignant diseases, tend to establish a seeming relationship between cancer and tuberculosis. The purpose of this paper is to clarify the difference in behaviour of the serum from cancer and tuberculosis patients in determining the type of " Index." The selection of tuberculosis serum was purely fortuitous. It arose from a desire for a " positive " control, as well as a negative, because the tuberculosis serum was supposed to resemble cancer in this test. From the experiments described here it would appear that a " positive " test encountered in tuberculosis does not apparently reflect the identical conditions of the blood serum responsible for a " positive " test in the case of a number of early, new and untreated cancer patients. This report is not an evaluation of the degree of accuracy of the method as a cancer diagnostic test.

### METHOD AND MATERIAL.

The test was carried out exactly as described by Huggins, Miller and Jensen (1949), except for a modification as suggested by them (private communication). This was the use of a wire loop, 11 to 12 mm. in diameter, that was quickly thrust into each test-tube after boiling the sera, to loosen the " tenacious " layer of film on top of the coagulum. The tube was inclined to approximately a 45° angle, to evaluate the degree of clotting or liquification. Difficulties in " reading " the degree of clotting were mainly encountered in the new cancer cases. That is, there appeared to be a higher incidence of transitional zones of the " shapeless viscous mass " type, than the clear-cut changes from clot to complete liquid that prevailed for the tuberculosis sera, the advanced treated cancer cases, and the majority of benign tumour subjects and normal healthy persons. Readings were done by two persons with agreement on identical tubes as to clots or complete liquification, and agreement within either of two tubes when considering the " shapeless viscous mass " type of coagulum. The factor which differentiates one tube from another is that of the micromoles of sodium iodoacetate. This, divided by the reciprocal serum protein per cent, serves to fix the type of " index," or :

$$\text{Iodoacetate Index (I.I.)} = \frac{0{\cdot}5 \times 2 \times \text{m}}{\%\ \text{protein}}$$

where 0·5 = ml. serum, m = micromoles of sodium iodoacetate in the nearest tube containing a firm clot preceding a tube in which the coagulum is a " shapeless viscous mass," or in the original state before boiling.

The serum protein determinations were carried out by the semi-micro Kjeldahl procedure recommended by Huggins, Miller and Jensen (1949). Every fifth determination was duplicated with an average agreement of $\pm$ 0·5 per cent. The procedure was frequently checked for accuracy for the recovery of 5·0 mg. quantities of nitrogen by carrying out a complete determination with 23·6 mg. quantities of anhydrous ammonium sulfate with recovery in duplicate being $\pm$ 0·02 to 0·03 mg. of nitrogen.

The source of the tuberculosis blood samples was the Harbor Hospital, Torrence, and the cancerous, benign neoplasia, and non-symptomatic blood samples were from our own clinic. All the tests reported here were done in the order in which they were received. For purposes of analysis the cases are regrouped as follows :

Group I consists of 46 biopsied, new, untreated cancer cases.

Group II consists of 36 advanced cancer cases in various stages of the disease.

Group III consists of 55 pulmonary tuberculosis cases, of which 11 were arrested.

Group IV consists of 92 biopsied benign growth-bearing patients.

Group V consists of 85 non-symptomatic individuals.

The thermocoagulation : per cent serum protein relationship of the various groups is represented graphically in Fig. 1.

It is necessary to comment on the clinical status of the various groups of subjects. All tests were performed after the diagnosis had been established. All the Group I patients were still in relatively good physical condition. None of these cases tested were followed up for repeat determinations, except the two comedocarcinomas, which were done before surgery. None of the treated cancer cases were H.M.J. tested (as we shall now refer to the test, since it was so designated by the authors) before treatment. The two myelomas were receiving nitrogen mustards ; the leucaemias, X-rays and/or transfusions ; some of the advanced cases, polysaccharides or chymotrypsin ; eight of the breast cancer cases, testosterone. None of the patients in Groups I and II received any special diet, except that the most advanced, uncontrolled cases were getting either plasma transfusions or protein hydrolysates of one kind or another. All of them received an iron-vitamin preparation. One of the 11 negative cases in Group III had an acid-fast sputum at the time the H.M.J. test was done.

### EXPERIMENTAL RESULTS.

Consideration of Table I shows the correlation of the serum protein per cent with the I.I., as representative of the various groups of cases. Only 24 out of 46 or approximately 52 per cent of the Group I cases demonstrated a positive H.M.J. (index less than 9·0), whereas 23 out of 36, or 63 per cent, of Group II, and 44, or 80 per cent, of Group III, also yielded a positive H.M.J. At first hand it would appear that the test was far more " efficient " in diagnosing tuberculosis than cancer. However, it will be noted that the I.I. was derived from protein

FIG. 1.—Distribution of iodoacetate indices and protein content of serum. The horizontal line represents the protein values in g. per 100 ml. serum, and the vertical line the corresponding indices, drawn from data. ⊕ = positive segment ; ⊖ = negative segment of correlation. ○ I, untreated cancer cases ; ● II, advanced cancer cases ; ■ III, pulmonary tuberculosis ; × IV, benign neoplasms ; ▼ V, non-symptomatic controls.

TABLE I.—*Summary of the Correspondence of Iodoacetate Index Range with the Total Protein Range.*

| Subjects. | Number of cases. | Iodoacetate index. | Total protein per cent. |
|---|---|---|---|
| Group I : Early cancer . . . . . | 24 . | 4·7– 8·8 . | 6·1–7·7 |
| ,, ,, . . . . . | 22 . | 9·0–13·8 . | 5·9–6·8 |
| ,, II : Advanced cancer, treated, uncontrolled . . . . . | 23 . | 6·2– 8·1 . | 4·4–5·7 |
| Advanced cancer, treated, controlled . . . . . . | 13 . | 9·5–12·2 . | 6·6–7·5 |
| ,, III : Active pulmonary tuberculosis | 44 . | 5·3– 7·3 . | 4·3–5·1 |
| Arrested pulmonary tuberculosis . . . . . | 11 . | 9·3–11·4 . | 6·3–7·2 |
| ,, IV : Benign neoplasia . . . | 78 . | 9·3–16·0 . | 5·9–7·6 |
| ,, ,, . . . | 14 . | 5·4– 8·4 . | 5·8–8·1 |
| ,, V : Non-symptomatic normals . | 83 . | 9·3–11·3 . | 6·2–8·2 |
| ,, ,, . | 2 . | 7·7– 8·9 . | 6·5–6·7 |

values well within the normal range for this procedure in Group I, while virtually the same I.I. range for Groups II and III was based upon a hypoproteinemia. However, in those instances where the result was a negative H.M.J., regardless of the clinical state, there was no such discrepancy in the serum protein values.

The observed variations in the properties of the serum are detailed in Table II, and Table III is a breakdown of the types of growth encountered with indices greater or less than 9·0.

A minor but interesting point emerging from our work is the fact that the numerical values of the positive range of indices for Groups I and II is not due

TABLE II.—*Frequency of Occurrence of Various Iodoacetate Indices in Different Conditions.*

| Iodoacetate. index. | Group I. Untreated cancer. 46 cases. (Per cent.) | Group II. Advanced cancer. 36 cases. (Per cent.) | Group III. Pulmonary tuberculosis. 55 cases. (Per cent.) | Group IV. Benign neoplasia. 92 cases. (Per cent.) | Group V. Non-symptomatic controls. 85 cases. (Per cent.) |
|---|---|---|---|---|---|
| 4·6– 5·0 | 26·1 | — | — | — | — |
| 5·1– 6·0 | 17·4 | — | 47·3 | 3·2 | — |
| 6·1– 7·0* | 4·3 | 27·8 | 29·1 | 6·5 | — |
| 7·1– 8·0 | 2·2 | 16·7 | 3·6 | — | 1·1 |
| 8·1– 9·0 | 6·5 | 19·4 | — | 5·4 | 1·1 |
| 9·1–10·0 | 15·2 | 13·9 | 7·3 | 18·5 | 26·2 |
| 10·1–11·0 | 10·9 | — | 7·3 | 24·0 | 2·4 |
| 11·1–12·0 | 10·9 | 19·4 | 5·4 | 14·2 | 26·2 |
| 12·1–13·0 | 6·5 | 2·8 | — | 16·3 | 39·4 |
| 13·1–14·0 | — | — | — | 4·3 | 3·6 |
| 14·1–15·0 | — | — | — | 5·4 | — |
| 15·1–16·0 | — | — | — | 2·2 | — |

* This range of indices includes the average values of the two comedocarcinoma cases.

to the severity or extensiveness of the malignant process. Despite the so-called " precancerous " nature of the two comedocarcinomas in Group I, the degree of " positiveness " was greater than for the lymphosarcoma and primary lung adenocarcinoma (I.I. between 8 and 9). The differential nature of the I.I. for the series of cases in Group IV merely reflects the ratio of concentration of iodoacetate as an inhibitor of coagulation for decreasing quantities of serum protein. There is no evidence that age distribution of the group tested played any significant role in the ratios obtained for the I.I.

### DISCUSSION

Analysis of the factors involved in the procedure showed that the coagulability of 52 per cent of the Group I sera is abnormal for entirely different reasons from the sera of Groups II and III. Evidence for this lack of homogeneity between the protein values and the degree of positive H.M.J., obtained for tubercular sera and the sera from new untreated cancer soon became apparent, i.e. the serum protein of the positive I.I. of the tubercular sera did not lie within the same range of protein per cent for the new cancer cases as for the tubercular. Furthermore,

TABLE III.—*Distribution of Iodoacetate Index for Malignant and Benign Tumours.*

| Group I: Malignant tumours. | Iodacetate index. <9·0. | >9·0. |
|---|---|---|
| Comedocarcinoma, duct glands | 2 | — |
| Sarcoma, breast | 2 | — |
| Squamous cell epithelioma lip | 1 | 2 |
| Adenocarcinoma nasopharynx | 1 | 1 |
| Lymphosarcoma | 1 | — |
| Malignant melanoma | 2 | — |
| Leukaemia, lymphatic | 1 | — |
| Adenocarcinoma ovary | 1 | — |
| ,, lung, bronchogenic | 1 | — |
| ,, cervix | 6 | 6 |
| ,, breast | 6 | 13 |
| | 24 | 22 |

| Group II. | Iodoacetate index. <9·0. | >9·0. |
|---|---|---|
| Multiple myeloma | — | 2 |
| Hodgkins | — | 3 |
| Adenocarcinoma thyroid | — | 1 |
| Lymphosarcoma, stomach | — | 1 |
| Squamous cell epithelioma mouth | 1 | — |
| Adenocarcinoma rectum | 2 | 3 |
| Lymphosarcoma, tonsil | 1 | 1 |
| Adenocarcinoma uterus | 4 | — |
| ,, cervix, with metastases | 6 | — |
| ,, ,, | 2 | 1 |
| ,, breast | 4 | 1 |
| ,, stomach | 2 | — |
| ,, duodenum, with metastases | 1 | — |
| | 23 | 13 |

| Group IV: Benign tumours. | Iodoacetate index. <9·0. | >9·0. |
|---|---|---|
| Basal cell carcinoma | 7 | 2 |
| Leukoplakia, mouth | 1 | 4 |
| ,, vagina | — | 5 |
| Cervical polyps | — | 10 |
| ,, fibrosis | 2 | 8 |
| Fibroid uteri | 1 | 17 |
| Pigmented verrucae | — | 12 |
| ,, naevus | — | 8 |
| Keratosis | 1 | 7 |
| Sebaceous cysts | — | 4 |
| Cystic breast | 2 | 1 |
| | 14 | 78 |

when we attempted to substitute a more rapid and less costly method for estimating the serum protein, such as a viscosimetric procedure (Kagan, 1938 ; Phelps and Van Slyke, 1943) our attempts met with failure when relating the I.I. to the Group I patients. In some the " positive " was altered to " negative," and in others the positive results became more or less positive. There was good agreement, however, for the I.I. values established by both Kjeldahl and falling-drop methods of protein estimations, for the remaining groups of cases. This led us to consider an " aberrant " or " combined-protein " as being responsible for the striking disagreement with the H.M.J. for the two types of disease.

The possibility that we were dealing with an increase in one of the normal fractions of the serum, the alpha globulins, reported to be increased in malignancy, led us to carry out the test on a sample of mucoprotein from cancerous serum, generously supplied by Dr. Richard Winzler, of the Department of Biochemistry, of the University of Southern California, and a " Bence Jones " protein, isolated from the urine of one of the two myeloma cases in this series. When adjusted to an equivalent of 7·0 g. per 100 ml. neither of these substances yielded a positive I.I. If, as others have observed, the degree of increase of alpha globulin is proportional to the severity of active tissue destruction, then apparently the alpha globulin plays no part in the Huggins test, either for advanced tuberculosis, which certainly involves active tissue destruction, or in the early and new untreated cancer cases in our study.

The question of the existence of modified serum protein molecules in various disease states, including malignancy, has been discussed recently by one of those whose published data established a close correlation between plasma viscosity, and the severity of the organic changes (Harkness, 1949). Added emphasis that we may be dealing with an altered protein in malignant tumours is given by the experimental observations of Hellwig (1949), who found that as a rule the size of spherical bodies in the form of clusters, short-chains and aggregates from tumour extracts when examined by electron microscope was exceedingly larger than that in benign or normal tissue. He concludes that these globules are not virus-like causative agents of cancer, but rather globular proteins, probable aggregates of the cytoplasmic globules due to an alteration in the colloidal state of the cancer cell. It is quite possible that this cytoplasmic material may be introduced into the blood serum and may play a role in accounting for the positive I.I. despite a normal range of serum protein in the 24 cases of Group I.

It is quite likely that those methods which measure the antiproteolytic activity (Clark, Cliffton and Newton, 1948), the rate of cell proliferation (Norris and Majnarich, 1948, 1949), the enzyme inhibiting or oxidation and reduction (Duboff and Hirshfeld, 1946 ; Savignac, Gant and Sizer, 1944) inhibition of a dye by human serum in early malignant disease involve the proteins or substances associated with the proteins of the serum.

Since the completion of this manuscript, Huggins, Jensen, Player and Hospelborn (1949) have published observations which support the theory of alteration of the serum protein molecule in malignancy, by employing the binding of a cationic dye. Only 44·7 per cent of their cases demonstrated a significantly abnormal effect.

Huggins and his colleagues have demonstrated a valuable and fundamental approach to the nature of these groups in health and disease. It is, however, well to record that their method suffers from one serious drawback—the large subjective element in the estimation of the coagulum. The task for the future is the elimination of this factor, perhaps, by measuring the degree of coagulation by an appropriate electronic procedure characterized by the electrical resistance or potential of the coagulum.

Can one explain the paradox of the 22 negative H.M.J. results in Group I ? This question should be studied from the following approach : If the 24 positive H.M.J. tests have a pattern of deformation in one direction, it is not inconceivable that the former 22 cases have a pattern of deformation in another direction that is not manifested in the test through the present combination of reagents. Furthermore, if the mechanism of action of this method is due to the increase of net negative charges on the protein molecule by the addition of sodium iodoacetate, then our results strongly suggest variations in the polar character of the protein molecule in the serum of certain phases of tumour development.

The magnitude of relationship between absolute protein per cent and the I.I. demonstrated by Group III serum does not reflect the magnitude of the effect, when applied to Group I. The term "false positive" cannot be applied, therefore, to such tuberculosis sera, in the same sense as the term "positive" is applied to that of early cancerous sera, since the term "positive" itself is not tenable on the same basis. The serum of individuals undergoing some type of degenerative process, appears to possess an admixture of some elements, the source of which we do not yet comprehend, but the presence of which has been demonstrated by measurable function of the thermocoagulation of protein in the presence of an extrinsic inhibitor, iodoacetate.

Tentatively, we are inclined toward the conclusion that abnormality in the blood serum of some cancer patients, unlike that of some tuberculosis blood with a corresponding I.I. (but not corresponding protein concentration), appears to be the effect of wholely or partially altered configuration of protein molecules as yet unidentified.

### SUMMARY.

1. Human sera from new untreated and advanced treated cancer, from advanced and arrested tuberculosis, benign growths, and non-symptomatic healthy persons were examined by a modified Iodoacetate Index Test.

2. There was a decrease in the Iodoacetate Index with corresponding decrease in the serum values for 100 per cent of the advanced tubercular and 63 per cent of the advanced cancer cases.

3. However, there was also a decrease of the Iodoacetate Index without a corresponding decrease in the serum protein for 52 per cent of the sera of new, untreated cancer, 17 per cent of the benign tumours and 1·2 per cent of the non-symptomatic controls.

4. The inhibited serum thermocoagulation phenomena involved in the Iodoacetate Index Test appears to be non-specific for active tuberculosis and advanced cancer, but appears to be specific in certain early or new untreated malignancies and benign tumour growths.

5. It is suggested that the use of the expression "false positive" or "pseudo-positive" be eliminated from the terminology of cancer tests.

The author wishes to tender his sincere thanks to Dr. Clyde K. Emery for making this work possible ; to Dr. Paul Rekers, now of the Institute for Medical

Research, Cedars of Lebanon Hospital, Los Angeles, for his constant advice and constructive criticism ; to Dr. William Bruckner, Director of the Harbor Hospital, Torrence, for the tuberculosis blood specimens, and his interest ; and to Peryl Ashton for her technical assistance.

### REFERENCES.

CLARK, C. G. C., CLIFFTON, E. E., AND NEWTON, B. L.—(1948) *Proc. Soc. exp. Biol. N.Y.*, **69**, 276.

DUBOFF, G. S., AND HIRSHFELD, S.—(1946) *Cancer Res.*, **6**, 57.

HARKNESS, J.—(1949) *Biochim. Biophys. Acta.*, **3**, 34.

HELLWIG, C. A.—(1949) *Arch. Path.*, **48**, 426.

HUGGINS, C., JENSEN, E. V., PLAYER, M. A., AND HOSPELBORN, V. D.—(1949) *Cancer Res.*, **9**, 753.

*Idem*, MILLER, G. M., AND JENSEN, E. V.—(1949) *Ibid.*, **9**, 177.

KAGAN, B. M.—(1938) *J. clin. Invest.*, **17**, 369.

NORRIS, E. R., AND MAJNARICH, J. J.—(1948) *Amer. J. Physiol.*, **153**, 483.—(1949) *Proc. Soc. exp. Biol. N.Y.*, **70**, 229.

PHELPS, R. A., AND VAN SLYKE, D. D.—(1943) *Bull. U.S. med. Dept.*, **71**, 66.

SAVIGNAC, R. J., GANT, J. C., AND SIZER, I. W.—(1944) *Amer. Ass. Adv. Sci.* Monograph on the Res. Conf. Cancer, p. 241.